# Development of a Low-Cost Portable EMG for Measuring the Muscular Activity of Workers in the Field

**DOI:** 10.3390/s23187873

**Published:** 2023-09-14

**Authors:** Mohamed Garouche, Ornwipa Thamsuwan

**Affiliations:** Department of Mechanical Engineering, École de Technologie Supérieure, Montreal, QC H3C 1K3, Canada; garouche.electronics@gmail.com

**Keywords:** muscle fatigue, low-cost electromyography, muscle injuries, musculoskeletal disorders

## Abstract

This study explores the development and validation of a low-cost electromyography (EMG) device for monitoring muscle activity and muscle fatigue by monitoring the key features in EMG time and frequency domains. The device consists of a Raspberry Pico microcontroller interfacing a Myoware EMG module. The experiment involved 34 volunteers (14 women, 20 men) who performed isometric and isotonic contractions using a hand dynamometer. The low-cost EMG device was compared to a research-grade EMG device, recording EMG signals simultaneously. Key features including root mean square (RMS), median power frequency (MDF), and mean power frequency (MNF) were extracted to evaluate muscle fatigue. During isometric contraction, a strong congruence between the two devices, with similar readings and behavior of the extracted features, was observed, and the Wilcoxon signed rank test confirmed no significant difference in the ability to detect muscle fatigue between the devices. For isotonic contractions, the low-cost device demonstrated behavior similar to the professional EMG device in 70.58% of cases, despite some susceptibility to noise and movement. This suggests the potential viability of the low-cost EMG device as a portable tool for assessing muscle fatigue, enabling accessible and cost-effective management of muscle health in various work scenarios.

## 1. Introduction

Muscle fatigue has been linked to various types of muscle injuries, including tears [1], and can lead to functional failure of muscles, thereby amplifying injury risk. If not managed correctly, it can also lead to muscle overwork and severe injuries [2]. Research has found that fatigued muscles have significantly less energy absorption capacity [3], suggesting muscle fatigue might be a substantial causative factor in muscle injuries. In sports and physical work activities, muscle fatigue can lead to spontaneous injuries due to reduced shock-absorbing capacity and changes in muscle contractile properties. Additionally, fatigue in specific muscles, such as quadriceps and hamstrings, impacts biomechanical factors related to anterior cruciate ligament injuries. Post-fatigue, a significant reduction is observed in knee joint extension and adduction moments and anterior bundle force [4]. Furthermore, fatigue in paravertebral muscles affects the ability to detect lumbar position changes in patients with recurrent or chronic lumbar disorders [5]. Muscle fatigue also negatively impacts the ability to distinguish different arm movement speeds, potentially leading to inefficient muscle use, increased muscle co-activation, and overwork. This shows that muscle fatigue has adverse effects on not only physical performance but also sensory perception and motor control, potentially increasing injury risk [6].

Muscle activity is an essential parameter to study for understanding muscle fatigue and preventing injuries in manual labor professions. However, accurate and ongoing evaluation of muscle activity in real-world work environments remains a challenge to be addressed. Currently available tools, such as professional electromyograms (EMGs), are often costly and require trained personnel for their usage and interpretation. This work thus attempted to overcome these challenges through the development of a low-cost, portable EMG.

This study acknowledged and built upon prior research into affordable EMG sensors. First, Del Toro et al.’s study [7] demonstrated the potential of a Myoware EMG module in detecting muscle fatigue using the Arduino Mega as a microcontroller, where they presented a good correlation between their low-cost device and a commercial EMG despite the presence of noise while performing isometric contractions. Additionally, Heywood et al. [8] highlighted the reliability of the same EMG module against a commercial system through tests with healthy participants. Moreover, Fuentes del Toro et al. [9] illustrated high concordance between another low-cost EMG sensor and a commercial system, despite signal interference and time lag. In addition, studies by Molina-Molina et al. [10] and Jang et al. [11] validated their affordable EMG systems through exercises, demonstrating excellent correlations with commercial sensors. Furthermore, Ahmed et al. [12] and Gehlot et al. [13] developed and validated affordable EMG devices to predict muscle fatigue prediction but limited their parameters to ones in the time domain. Lastly, Bawa and Banitsas [14] validated an affordable MyoTrack EMG sensor through dynamic exercises, underlining excellent agreement between the systems. Overall, even though there have been some explorations in the use of a Myoware EMG, the research is rare and relatively limited in terms of the parameters of interest and activities used for their validation.

In this study, a portable EMG was designed with the aim to be more economical and practical for field usage. Since a low-cost system could suffer from issues of noise and inaccuracy due to the absence of high-quality analog filters and amplifiers, such as those used in commercially available research systems, it often requires advanced algorithms such as particle swarm optimization [15], meta-heuristics [16], and machine learning such as support vector machine [17]. However, because the intention for our device was to take basic EMG readings that could detect a sign of muscle fatigue, it was expected to demonstrate acceptably similar muscle fatigue indicators to those provided by a research-grade device. To address this issue, the rates of changes in EMG muscle fatigue parameters, including the root mean square (RMS) amplitude, the median power frequency (MDF), and the mean power frequency (MNF) [18], obtained from our device were compared to a professional EMG device.

In this manuscript, the methods are divided in two main sections describing (1) the modules of the low-cost device and (2) its validation by comparing the ability of the device to detect muscle fatigue as compared to a commercial, research-grade device. The results of the validation during isometric and isotonic contractions are then presented, followed by discussion and analysis of the device’s limitations according to the results and the background knowledge from previous studies.

## 2. Materials and Methods

### 2.1. Low-Cost EMG Device

**Figure 1:**
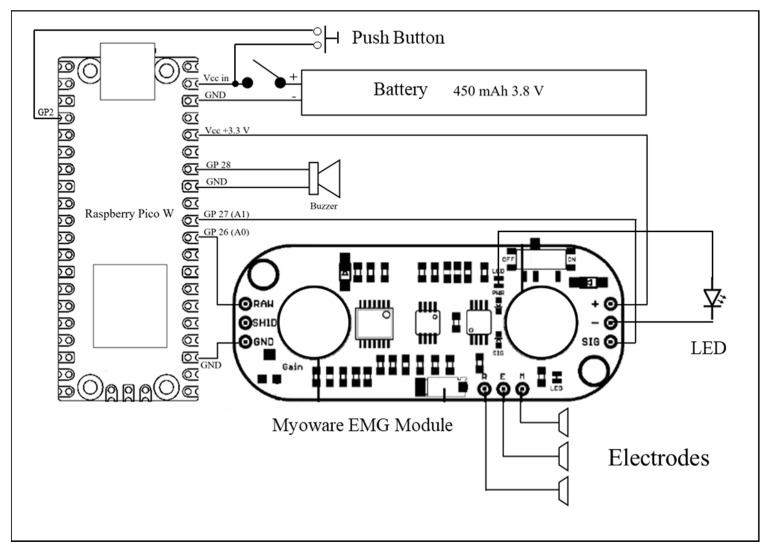
Diagram of the low-cost EMG system.

The system consisted of a Myoware EMG module (Advancer Technologies, Milford Center, OH, USA) connected with a Raspberry Pico board (Raspberry Pi, Cambridge, United Kingdom) through the utilization of three distinct wires (Figure 1):

1. A Ground wire, serving as a conduit for grounding, established a linkage between the module’s ground potential and that of the microcontroller.
2. A Vcc wire served as a channel for power transmission, interfacing the power output of the microcontroller with the power input of the module.
3. An additional wire established a linkage between the “Raw” output, synonymous with the unprocessed signal, and the analog input residing on pin 26 of the microcontroller.

The employment of the RAW output, in contrast to the SIG output, enabled the capacity to manipulate the original signal. This augmented maneuverability facilitated diverse feature extraction methodologies. The outcome of this manipulation thus allowed for heightened precision in control and a more profound customization of the data processing workflow. Such adaptability was of paramount significance within the context of our ongoing investigation into the quantification of muscle fatigue.

It should be noted that no additional transducer was included in the system, but only the onboard 12-bit ADC of the Raspberry Pico was used. In addition to the Myoware onboard analog filter from 10 to 450 Hz, our system used a digital filter of 20–450 Hz.

Incorporated within the system was an auditory component in the form of a buzzer. This acoustic element served a dual purpose: firstly, to apprise the user of the system’s successful connection to the client device, and secondly, to indicate the commencement of the recording session.

The system employed Python code to establish communication with a client device, encompassing options such as computers, tablets, and smartphones. This interaction was facilitated through the utilization of the TCP protocol, leveraging the integrated Wi-Fi chip situated on the Raspberry Pico board.

Since the Raspberry Pico has two CPU cores, the CPU usage was divided by using a low-level threading API “_thread” on Mycropython. The first core was dedicated to reading the data from the Myoware module at the sampling rate of 2000 Hz. The second core was responsible for establishing the connection with the device and sending the data collected via a socket protocol using the integrated Wi-Fi chip (2.4 GHz) with a range of approximately 45 m indoors and 90 m outdoors.

The comprehensive cost of the device was approximately USD 350, including not only the essential components but also auxiliary items such as a plastic enclosure designed to house the entirety of the system, alongside the inclusion of electrodes necessary for its operation.

### 2.2. Validation for Muscle Fatigue Detection

This section serves as a validation of the device. The low-cost device developed in-house (referred to as Myoware) was compared to a research-grade device. As this study focused on the ability to detect signs of muscle fatigue, the parameters of interest were the Spearman correlations between RMS and time, MDF and time, and MNF and time. This following section describes the human subject experiment conducted for this validation.

#### 2.2.1. Participants

A total of 34 healthy participants, comprising 20 men and 14 women, were recruited for this experiment from a local gym club. Prior to the experiment, the protocol and potential risks involved in this study were explained to all participants, and informed consent was obtained. Table 1 summarizes the personal information gathered from the participants. The test statistics and *p*-values showed no statistically significant difference in age, frequency of weekly trainings, and BMI among the male and female participants.

#### 2.2.2. Instrumentation

In preparation for data acquisition, the forearm of the participant’s dominant hand was shaved. This was performed to facilitate effective attachment of the EMG electrodes (271S, Noraxon, Scottsdale, AZ, USA). Two sets of electrodes were placed on each participant’s forearm: one set was from our low-cost device, and the other set was from a research-grade EMG system that served as our validation tool, consisting of a datalogger (MWX8, Biometrics Ltd., Newport, UK) equipped with a 32-bit microcontroller, a 14-bit analog-to-digital converter (ADC), and dry electrodes with a built-in amplifier (SX300, Biometrics Ltd., Newport, UK). EMG data were collected at a sampling frequency of 2000 Hz.

**Figure 2:**
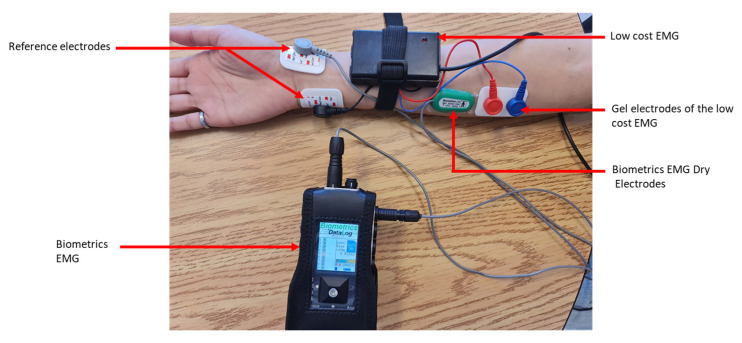
General arrangement of the electrodes in the two systems.

The electrodes were carefully positioned above the flexor carpi radialis muscle. It is worth noting that the orientation of the electrodes was meticulously adjusted to align with the direction of the muscle fibers. This was a crucial step as the alignment of the electrodes with the muscle fibers can significantly influence the accuracy of the EMG readings. In addition to the primary electrodes, reference electrodes were also used in this experiment. The reference electrodes were placed on a bony area of the wrist. This placement helped to minimize interference from electrical noise and other factors that could affect the accuracy of the EMG measurements (Figure 2). The SENIAM protocol [19] was applied for determining the position of the electrodes on the muscle bundle as well as the inter-electrode space of 20 mm in both sets of electrodes (Figure 2).

#### 2.2.3. Data Collection

The participants were solicited to perform their regular warm-up to minimize any risk of muscle injury during the experiment. Then, the experiment took place in three main phases while the EMG signals were recorded:

Phase 1: Three sequences of maximal voluntary contractions (MVCs) using a handheld dynamometer were recorded. Participants squeezed the handle of the dynamometer (grip force) as hard as possible for 8 s. This step was repeated two more times with a two-minute break between each sequence. In each recording, the root mean square (RMS) amplitudes within 100-millisecond windows through the 8-second recording were visualized. Only the plateaus after the ramp and before the drop were cropped. Then, the median of this RMS was calculated. The MVC was determined as the maximum value of these three medians. This phase was essential for normalizing EMG signals, as it allowed for comparing the measurements of the two EMG systems among different participants.

Phase 2: After a five-minute rest, participants performed isometric contractions using the same device. They were asked to apply 50% of their maximum force, determined during the MVC, to squeeze the dynamometer handle and try to maintain this force for 90 s. It should be noted that the participants could visualize the force applied to the dynamometer handle thanks to a mirror placed in front of them during this step.

Phase 3: After another five-minute rest, participants were asked to perform isotonic contractions for 90 s using the same device. To obtain cyclic rather than random isotonic contractions, our application generated a sound every second for the participant to squeeze the dynamometer handle when they heard a beep, and to release it when a different beep was heard. The number of these contractions was determined by the participant’s fatigue level. If the participant wished to stop contractions before the 90-second duration, this phase was then considered complete.

**Figure 3:**
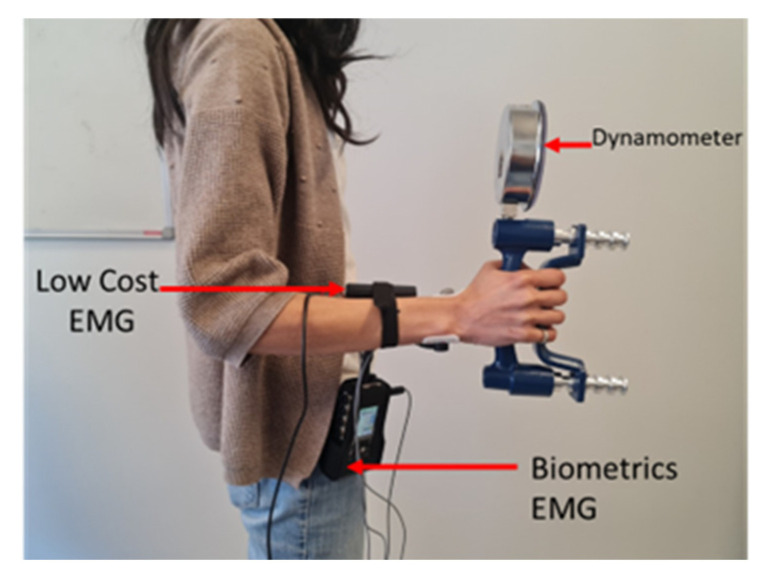
Participant’s position and arrangement of the two systems.

During Phases 2 and 3, the participant was invited to press a push-button connected to the low-cost EMG system at the moment they felt fatigue in the forearm, i.e., when they started to feel discomfort as a result of squeezing the dynamometer and they could no longer maintain the same squeezing force. The obtained signals were normalized with a standard protocol in real-time using the MVC data collected during the first phase. Throughout these phases, the participants were in a standing position at the height of the mirror, with elbows locked at a 90-degree angle facing a mirror (Figure 3).

**Figure 4:**
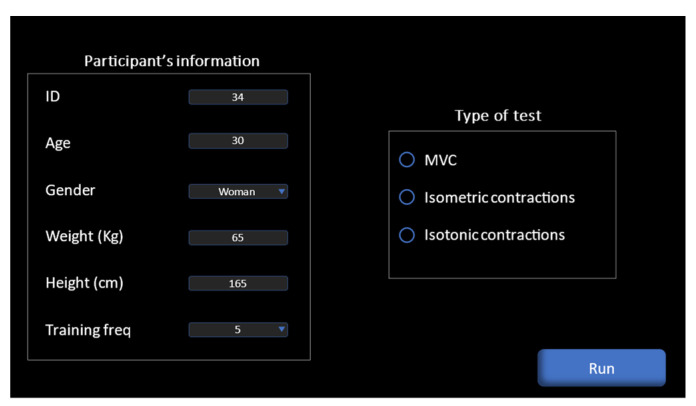
Interface of our low-cost system.

Our system was equipped with a dedicated application that operates on computers and smartphones, where a connection was made via Wi-Fi, for the purpose of controlling the recording of the signal and real-time monitoring of muscle activity. To start recording, the participant’s identification number, age, height, and weight; the frequency of their workouts, and the type of contraction were input, and finally, the recording start button (“Run” in Figure 4) was clicked. Regarding the Biometrics device, the recording was made by simultaneously pressing the record button on the dedicated software.

Our application was programmed to stop automatically after 8 s in the case of recording maximal voluntary contractions, or 90 s in the case of an isometric or isotonic contraction. Conversely, in the Biometrics device, the users had to manually click the stop button, and it did not distinguish the type of recording. Data were immediately transferred in real-time from the low-cost sensor to our signal processing application. In the Biometrics device, the memory card had to be removed and the data had to be transferred to the computer. Then, the software dedicated to the device needed to be used for exporting the data to a CSV file for analysis.

#### 2.2.4. Data Processing

The signals obtained from both devices were subjected to a filtering procedure using a 4th-order Butterworth band-pass filter in the range of 20 to 450 Hz. Subsequently, the harmonics of 60 Hz frequencies were eliminated using a Butterworth notch filter. Regarding our device, this filtering procedure was implemented at the time of data reception. The resulting signal was instantly visualized graphically within our application.

In this study, three characteristics of the EMG signal were extracted. One of these characteristics came from the time domain, namely normalized RMS, while the other two, MDF and MNF, were derived from the frequency domain. These are generally considered as principal indicators of local muscle fatigue [18]. To achieve this, we used two strategies.

The first, intended for isometric contractions, consisted of calculating these characteristics over a distant window of one-second size without overlap for the entire signal resulting from these contractions. The second, intended for isotonic contractions, was different as we were faced with isotonic contractions, meaning the EMG signals changed over time, just like the muscle length. Therefore, the signal could not simply be analyzed by applying a fast Fourier transform (FFT). Instantaneous mean and median frequencies (IMNF and IMDF) were introduced to meet this requirement using time-frequency approaches such as the short-time Fourier transform (STFFT) [20]. A method similar to that of Georgakis et al. (2003) [21] was used to calculate the average of the IMNF and IMDF. Indeed, the use of the average of the IMDF and IMNF can help to obtain a good representation of the signal. The fluctuations of the instantaneous values of the IMNF and IMDF were smoothed by averaging values over a certain time window with the size of 100 samples, determined after several trials. This helped to reduce noise and short-term variations of the signal, allowing us to observe more stable and consistent trends in the signal.

#### 2.2.5. Statistical Analysis

Considering the non-normal distribution of our data as previously verified through the application of the Shapiro–Wilk test to confirm non-normality, non-parametric statistical tests were employed. Our initial approach entailed the computation of the Spearman rank correlation coefficient, considering the three distinct characteristics RMS, MDF, and MNF in relation to time. This was carried out to determine whether the two devices could detect temporal variations in the characteristics, thereby enabling the detection of indicators of muscle fatigue. Lastly, Wilcoxon rank-sum tests were performed on the Spearman coefficients of the characteristics RMS, MDF, and MNF over time to determine if the correlations were similar between the two devices.

## 3. Results

### 3.1. Muscle Fatigue Detection in Individuals

#### 3.1.1. Isometric Contractions

Muscle fatigue was detected through the changes in RMS, MDF, and MNF over time. Figure 4 illustrates an example of the three characteristics extracted from the RMS, MDF, and MNF of the filtered signal from the isometric contractions by one participant obtained from the two devices. On the left (A), the characteristics extracted from the signal collected with the low-cost device showed great similarity with the characteristics from the signal collected from the Biometrics device, on the right (B). An evident increase in the normalized RMS value (in blue) and decreases in the two frequencies (MDF in grey and MNF in green) over time could be observed for both devices. The red dotted line represents the moment when the participant pressed the fatigue button as they felt their forearm muscle could no longer provide the necessary force to maintain pressure on the dynamometer handle.

There were no remarkable changes between the moment when the participant perceived the onset of fatigue and the characteristics observed for most participants. However, for some participants, when they pressed the fatigue button, their attention was momentarily diverted, resulting in a slight drop in the RMS before it rebounded.

**Figure 5:**
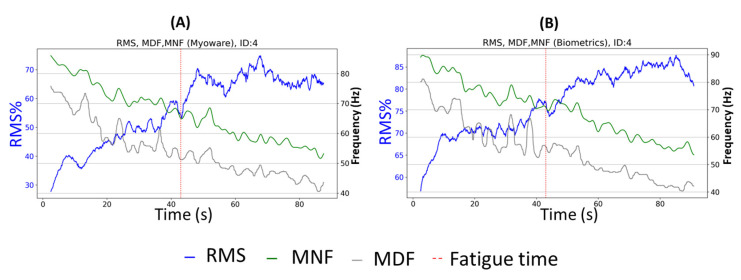
Comparison between the characteristics extracted from the two devices during the isometric contraction of one participant: (**A**) represents the signal from the low-cost device (Myoware), and (**B**) is the signal from the research-grade device (Biometrics).

**Figure 6:**
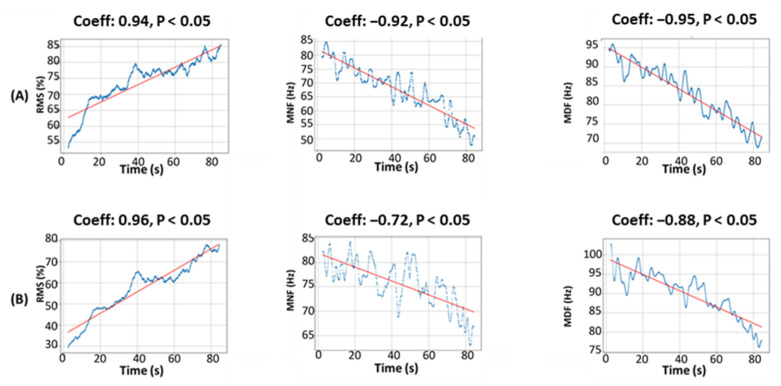
Correlation analysis results between signal characteristics and time during the isometric contraction of one participant: (**A, left**) RMS–time Myoware; (**A, center**) MDF–time Myoware; (**A, right**) MNF–time Myoware; (**B, left**) RMS–time Biometrics; (**B, center**) MDF–time Biometrics; (**B, right**) MNF–time Biometrics.

Figure 5 presents an example in one participant of the results of the Spearman correlation test for the three features and time steps measured from the two devices. According to the left diagram in Figure 6A, the correlation test between the RMS and time variables yielded a *p*-value below the significance threshold of 0.05; therefore, the null hypothesis of no significant correlation was rejected. In other words, the results indicated a significant positive correlation between the two variables. This conclusion was reinforced by the Spearman rank correlation coefficient of 0.94, which indicated a strong positive correlation between the RMS and time. According to the results of analysis (A, center), the correlation test between MDF and time produced a *p*-value below the significance threshold of 0.05, suggesting a significant correlation between the two variables. This conclusion was also reinforced by the Spearman rank correlation coefficient of −0.92, which signified a strong negative correlation between MDF and time. Similarly, the analysis in (A, right) revealed that MNF and time also had a significant negative correlation, with a *p*-value less than 0.05 and a Spearman rank correlation coefficient of −0.95. These results pointed to a strong negative correlation between the two variables. The results obtained for the signal characteristics recorded in the Biometrics device, represented by the letter (B), performed similarly to those obtained for the characteristics collected from the low-cost device.

#### 3.1.2. Isotonic Contractions

The apparition of muscle fatigue during isotonic contractions was analyzed based on the IMDF and IMNF calculated using the short-time Fourier transform (STFFT). Rapid fluctuations of both IMDF and IMNF could be observed, making it difficult to interpret these results. The frequency-domain features did not show significant behavior upon our initial visual inspection of the graphs. After calculating the average of the IMDF and IMNF over non-overlapping 1-second windows, clearer reading features could be defined, i.e., a drop in both IMDF and IMNF.

It was noticeable that the IMDF and IMNF were generally higher in our device than in the Biometrics device. The Spearman rank correlation coefficients were calculated to evaluate the correlation between time and RMS, average IMDF, and average IMNF gathered from both the low-cost device and the Biometrics device. In most cases (26 cases or 76.47%), one could observe a similar behavior of features in both devices. In other cases (eight cases or 23.52%), different profiles between the devices were observed; for example, when a feature increased in one device, we noticed an opposite result on the other.

### 3.2. Group Comparison of the Fatigue Detection between the Two Devices

#### 3.2.1. Isometric Contractions

Overall, we found that for the majority of participants, there were moderate to strong positive correlations (median = 0.73, Q1 = 0.46, Q3 = 0.91) between the RMS and time for both devices, as well as moderate to strong negative correlations between the two frequency-domain characteristics (median = −0.83, Q1 = −0.93, Q3 = −0.61 for MNF; median = −0.76, Q1 = −0.90, Q3 = −0.56 for MDF) and time. This suggests that we observed muscle fatigue in both devices and that most of the characteristics from the time and frequency domains exhibited comparable behavior in both devices.

The Wilcoxon rank-sum test between the coefficients of the Spearman correlations during isometric contractions yielded the results shown in Table 2. With *p*-values greater than 0.05, there was no statistically significant difference in the ability to detect muscle fatigue between the two devices.

#### 3.2.2. Isotonic Contractions

Similarly to the isometric contraction results, the Wilcoxon rank-sum test results for the parameters collected using the two devices during isotonic contractions (Table 3) suggested the absence of a statistically significant difference between the coefficients corresponding to the two devices (*p*-value greater than 0.05). In other words, the Spearman correlation coefficients of the devices did not exhibit notable differences, and the characteristics observed for these devices appeared to behave similarly in most cases.

Overall, 50% of participants did not press the fatigue signaling button during the 90-second isotonic contractions. For the remaining participants who did press the button, there was uncertainty regarding their subjective fatigue state. Specifically, these participants expressed confusion between the discomfort sensation induced by the manipulation of the dynamometer handle and the onset of actual muscle fatigue in their forearm.

**Figure 7:**
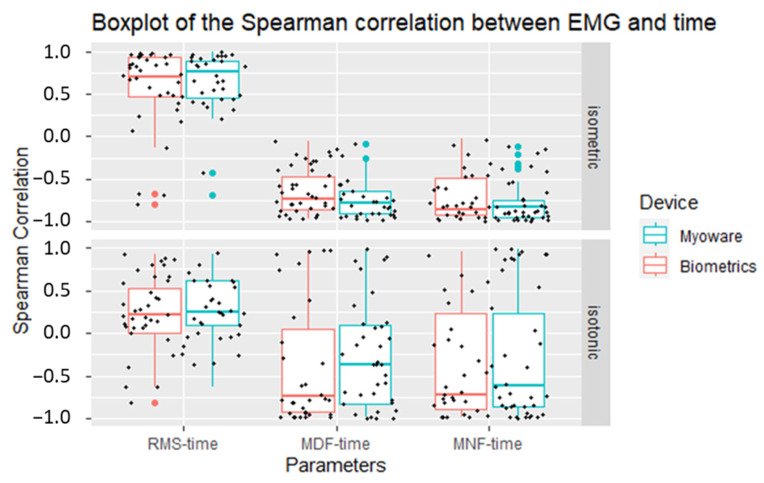
Boxplots of Spearman correlations between EMG parameters and time.

Finally, when examining the dispersion of the correlations between these parameters and time, unlike the Spearman correlations at the isometric contraction when muscle fatigue was detected more easily (Figure 7, top: mostly positive RMS–time correlation and negative MDF–time and MNF–time correlations), it was not clear whether muscle fatigue appeared during the isotonic contraction (Figure 7, bottom: Spearman correlation spreading from −1 to 1).

## 4. Discussion

As the results demonstrated, during the isometric contractions, both devices showed notable similarity in the behavior of the evaluated parameters. The application of the Spearman correlation coefficient revealed significant positive correlations between the RMS value and time for each of the two devices. This phenomenon of muscle fatigue onset was also observed for MDF and MNF, where strong negative correlations in relation to time were recorded. The application of the Wilcoxon rank-sum test corroborated the absence of a statistically significant difference between the correlation coefficients of the same characteristics collected using the two devices. This suggested a similar ability to detect muscle fatigue during these contractions within the context of our study.

However, we identified some divergences in the behavior of RMS, MDF, and MNF over time. These divergences were primarily related to two participants in the Spearman correlation results who were unable to continue providing 50% of their maximum effort throughout the entire experiment. During these divergences, we observed a drop in RMS and a rise in the MDF and MNF, showing the typical signs of fatigue. It is essential to note that during these divergences, both devices produced similar readings and demonstrated comparable behavior for the three observed characteristics. This reinforced their consistency in terms of performance and sensitivity to the variations of the studied features.

In a more detailed perspective on the isotonic contractions, a similarity in profiles for the parameters studied was observed in approximately 76.47% of the cases between the two devices. The divergences (23.52%) might be attributed to the variation of muscle contraction velocity performed by different participants, which was not systematically part of the protocol. Specifically, it was observed that certain participants produced more pronounced contractions than others in response to the sound signal indicating the onset of the contraction. These abrupt movements may have led to higher or different readings on the devices, thereby contributing to the observed divergence. Another plausible hypothesis for this divergence could be related to the nature of the contraction, given that this is the only parameter that underwent a change in comparison with the isometric contractions. Indeed, when a muscle contracts and then relaxes, the innervation zone and the tendon region can slide relative to the skin and the detection electrodes. According to the studies of [22], this sliding effect could lead to modifications in the EMG amplitude of more than 200%. This could be falsely interpreted as an increase in muscle activity. Moreover, other research [23] confirmed EMG amplitude fluctuations with variations in knee angles.

Furthermore, muscle contraction can induce a crosstalk phenomenon [24], which is interference between electrical signals originating from different muscles. This observation suggested that changes in muscle length can influence the quality and integrity of the collected electrical signal. Additionally, the non-identical position of the electrodes could have led to divergent measurement readings depending on the specific muscle area where each electrode was located during contraction. This situation could be attributed to variations in muscle activity and electrophysiological properties within the different areas covered by the electrodes.

We also observed that the frequencies recorded during the isotonic contractions exceeded those generated by the isometric contractions. It is possible that this divergence can be attributed to the fact that 50% of the subjects did not express symptoms of muscle fatigue. A plausible explanation for this phenomenon could be the relatively brief duration of the experiment. This suggests the absence of the downward trend of frequencies typically perceived in the context of isometric contractions, thereby allowing the maintenance of frequencies at higher levels. It is worth noting that the relaxation phase during isotonic contractions could also contribute to the increase in frequencies or their maintenance at higher levels due to several factors. These factors include a reduction in the synchronization of motor units, variations in length and muscle impedance following relaxation, and a redistribution of muscle fiber recruitment, allowing recovery of those that participated in muscle contraction during the contractile phase [25]. We must also take into account the difference in signals between individuals during the two types of contractions, as each individual has their own morphology and training routines. Indeed, not all individuals train in the same way, nor with the same intensity or frequency. These individual variations could thus influence muscle responses during contractions, adding another layer of complexity to our analysis.

Regarding accuracy and resolution, we expected that the Biometrics EMG system would be superior to our device because it was equipped with a more efficient analog-to-digital converter (ADC). The primary function of an ADC is to transform an analog signal, such as the electrical activity measured by an EMG device, into a digital signal that can be processed and analyzed by a computer. The ADC presented on the Biometrics device was 14 bits, while our device had a 10-bit one. A 10-bit ADC can represent 2^10^—i.e., 1024—distinct numerical values, whereas a 14-bit ADC can represent 2^14^—i.e., 16,383—distinct numerical values. The higher ADC bit depth resulted in a more accurate representation of the analog signal, allowing for measuring a broader range of signal amplitudes. This provided a superior resolution compared to the 10-bit ADC, thus enabling a more detailed digital representation of the analog signal [26]. This was particularly useful for capturing slight variations in the EMG signal that might have been missed with a lower-resolution ADC.

We also observed that the MNF was higher on our device compared with the Biometrics device. This is indicative of our device’s sensitivity to noise, given that the MDF was more stable in the presence of noise, unlike the MNF, and was much more sensitive to muscle fatigue than to noise [18]. This observation was explained by the fact that the lower-resolution ADC of our device resulted in a lower SNR, while the higher bit depth ADC presented on the Biometrics EMG benefited from a better SNR. This gave a better distinction between the true EMG signal and any potentially present noise in the signal. Consequently, this translates into sharper and more precise measurements [27].

**Figure 8:**
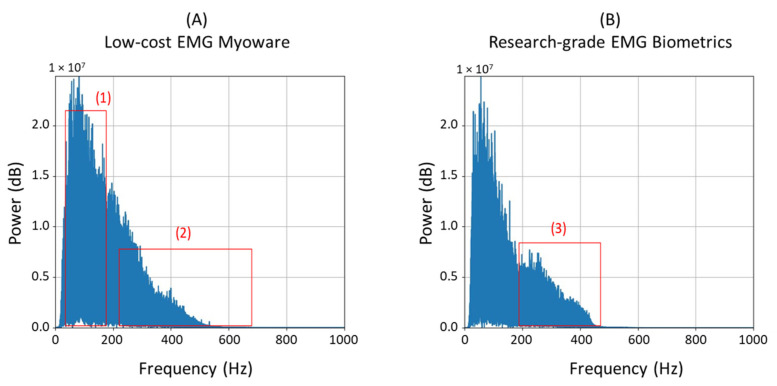
Differences between frequency spectra (isotonic contractions). (**A**) Myoware; (**B**) Biometrics.

The type of electrodes and their placement could have also impacted the overall performances of the devices. Since our device used flexible gel patch electrodes, this probably allowed their deformation, thus generating additional noise from skin movements. The length of the wires used could have also allowed for the collection of noise coming from various sources such as lighting, which could also explain the difference in the frequency spectrum graphs for the two devices during isotonic contractions for certain participants. This effect can be observed on the frequency spectrum during isotonic contractions for certain participants in Figure 8 (in rectangles 1 and 2 of part A).

In Figure 8, it is also notable that for most cases (20 in 34 cases), the Biometrics device presented richer frequency components in the range between 200 and 400 Hz (B, rectangle 3) compared to our device (A, rectangle 2), which seemed to have more frequency components in the range between 100 and 200 Hz (rectangle 1). This difference could also be related to the noise captured by our device, as the quality of the analog filter also played an important role in producing clear signals.

## 5. Conclusions

We developed a low-cost EMG device, consisting of a Myoware EMG sensor and a Raspberry Pico microcontroller, to carry out basic EMG data acquisition and potentially detect localized muscle fatigue. We validated the equipment with a research-grade device (Biometrics) through a field experiment involving 34 healthy participants. Our research hypothesis was confirmed, indicating that, despite being sensitive to movements and noise, our device ultimately managed to provide suitable readings of EMG parameters in the time and frequency domains.

The ability of our device to detect muscle fatigue could contribute to fields related to sports, ergonomics, and prosthetics. An automated system capable of detecting the onset of muscle fatigue in real-time could be useful in sports scenarios, where fatigue can lead to injuries. Such a system would guide the user in their training by acting as an alert before reaching a dangerous level, thus avoiding unnecessary muscle strain likely to cause injuries. This system can also be extended to applications in occupational health and ergonomics, particularly where there is a risk of work-related musculoskeletal disorders. Localized muscle fatigue in the workplace can lead to injuries—for example, in tasks that cause high static muscle activity. Likewise, in ergonomics, such a system can contribute to identifying problems before the onset of injuries. By making EMG more accessible to a larger number of workers, this research could contribute to the better monitoring of muscle fatigue and, thus, to the prevention of injuries in the workplace. Finally, our study could serve as a basis for future research in the field of low-cost wearable medical technology.

## Figures and Tables

**Table 1:** Summary of the collected personal information.

Gender	Age	Training per Week	BMI
Men	32.0 ± 4.9	1.5 ±1.7	23.9 ± 3.9
Women	32.3 ± 5.2	1.7 ± 1.9	24.2 ± 3.8
t statistics	−0.174	−0.315	−0.224
*p*-value	0.86	0.75	0.82

**Table 2:** Results of the Wilcoxon rank-sum test between the Spearman coefficients (isometric contractions).

	RMS Coefficient	MDF Coefficient	MNF Coefficient
Stat test W	217.5	208.5	228.5
*p*-value	0.54	0.19	0.70

**Table 3:** Results of the Wilcoxon rank-sum test between the Spearman coefficients (isotonic contractions).

	RMS Coefficient	IMDF Coefficient	IMNF Coefficient
Stat test W	189.0	252.0	233.5
*p*-value	0.16	0.61	0.56

## Data Availability

The data presented in this study are available on request from the corresponding author. The data are not publicly available due to the large size of the data in .wav format.
